# Pontos-Chave na Estimativa dos Parâmetros Hemodinâmicos Vasculares Pulmonares

**DOI:** 10.36660/abc.20230579

**Published:** 2024-03-12

**Authors:** Yusuf Ziya Şener

**Affiliations:** 1 Hacettepe University Faculty of Medicine Internal Medicine Department Ankara Turquia Hacettepe University Faculty of Medicine – Internal Medicine Department, Ankara – Turquia

**Keywords:** Pressão Propulsora Pulmonar, Anemia, Ecocardiografia, Cateterismo Cardíaco

**Prezado Editor,**

Li com grande interesse o manuscrito comparando as medidas hemodinâmicas do leito vascular pulmonar obtidas por ecocardiografia transtorácica e cateterismo cardíaco invasivo direito. Os autores relataram que, embora os resultados de ambas as intervenções diagnósticas se correlacionem, quase metade das medidas ecocardiográficas da pressão sistólica da artéria pulmonar e da pressão atrial direita são precisas.^1^

O status do volume é crucial na avaliação dos parâmetros hemodinâmicos vasculares pulmonares. A medida ecocardiográfica é realizada em ambulatório; entretanto, o cateterismo cardíaco é realizado em uma unidade de cateter. Os pacientes submetidos ao cateterismo cardíaco geralmente são aconselhados a não comer ou beber nada algumas horas antes do procedimento. Além disso, pacientes com sobrecarga de volume podem ter sido tratados com diuréticos para que tolerassem ficar deitados durante o procedimento. Parâmetros hemodinâmicos como pressão capilar pulmonar, pressão arterial pulmonar e pressão atrial direita são afetados pelo status do volume.^2^ O status do volume pode ter mudado durante o intervalo de três meses entre a avaliação ecocardiográfica e o cateterismo cardíaco.

O nível de hemoglobina tem impacto na pressão da artéria pulmonar. A anemia grave causa vasoconstrição pulmonar e insuficiência cardíaca de alto débito.^3^ Por outro lado, a deficiência de ferro que resulta em anemia se desenvolve com base em vários mecanismos na hipertensão pulmonar.^4^ Portanto, o círculo vicioso entre hipertensão pulmonar e anemia deve ser levado em consideração na avaliação dos parâmetros hemodinâmicos vasculares pulmonares.

Para concluir, embora tenha sido relatado que o intervalo de três meses entre as duas modalidades diagnósticas foi uma limitação, deve-se notar que o status do volume e os níveis de hemoglobina podem ter sido diferentes e possivelmente afetaram os resultados durante as duas medições distintas.
