# Supplementary material for: Pontos-Chave na Estimativa dos Parâmetros Hemodinâmicos Vasculares Pulmonares
Source: Arq Bras Cardiol. 2024 Mar 12;121(3):e20230579. [Article in Portuguese] doi: 10.36660/abc.20230579 (PMC12092060; doi:10.36660/abc.20230579)
Supplement: Translation

# Translation: Key Points in Estimating Pulmonary Vascular Hemodynamic Parameters

**Authors:** Yusuf Ziya Şener

**Affiliations:** 1 Ankara Turkey Hacettepe University Faculty of Medicine – Internal Medicine Department, Ankara – Turkey

**Keywords:** Pulmonary Wedge Pressure, Anemia, Echocardiography, Cardiac Catheterization

**Dear Editor,**

I have read the manuscript comparing the hemodynamic measurements of pulmonary vascular bed obtained from transthoracic echocardiography and invasive right heart catheterization with great interest. The authors reported that although the results of both diagnostic interventions correlate with each other, nearly half of echocardiographic measurements of systolic pulmonary artery pressure and right atrial pressure are accurate.^1^

Volume status is crucial in the assessment of pulmonary vascular hemodynamic parameters. Echocardiographic measurement is performed in an outpatient clinic; however, heart catheterization is performed in a catheter unit. Patients undergoing heart catheterization are usually advised not to eat or drink anything a few hours before the procedure. Moreover, patients with volume overload might have been treated with diuretics in order to tolerate lying down during the procedure. Hemodynamic parameters such as pulmonary capillary wedge pressure, pulmonary artery pressure, and right atrial pressure are affected by volume status.^2^Volume status might have changed during the three-month interval between echocardiographic assessment and heart catheterization.

Hemoglobin level has an impact on pulmonary artery pressure. Severe anemia causes pulmonary vasoconstriction and high-output heart failure.^3^On the other hand, iron deficiency resulting in anemia develops on the basis of several mechanisms in pulmonary hypertension.^4^Therefore, the vicious cycle between pulmonary hypertension and anemia should be taken into account in the assessment of pulmonary vascular hemodynamic parameters.

To conclude, although it was reported that the three-month interval between the two diagnostic modalities was a limitation, it should be noted that volume status and hemoglobin levels might have been different and possibly affected the results during the two distinct measurements.
