# Supplementary material for: Pontos-Chave na Estimativa dos Parâmetros Hemodinâmicos Vasculares Pulmonares
Source: Arq Bras Cardiol. 2024 Mar 12;121(3):e20230579. [Article in Portuguese] doi: 10.36660/abc.20230579 (PMC12092060; doi:10.36660/abc.20230579)
Supplement: Related texts

# Reply: Carta-resposta

**Authors:** Camila Farnese Rezende, Eliane Viana Mancuzo, Maria do Carmo Pereira Nunes, Ricardo Amorim Corrêa

**Prezado Editor,**

Gostaríamos de agradecer os comentários muito pertinentes e esclarecedores do leitor sobre as variáveis que têm o potencial de explicar discordâncias entre as estimativas ecocardiográficas (ECO) das pressões sistólicas do átrio direito (PAD) e da artéria pulmonar (PSAP) e aquelas obtidas por cateterismo direto das câmaras cardíacas direitas e da artéria pulmonar (CCD).

Concordamos com os comentários. O estado do volume intravascular pode variar ao longo de um intervalo de três meses, especialmente em pacientes ambulatoriais. É sabido que esta avaliação pelo ECO é complexa, dependendo dos parâmetros da veia cava inferior e até mesmo da medida invasiva da pressão venosa central, o que não é feito rotineiramente na prática clínica. Embora esta seja uma limitação do estudo, na prática clínica o volume plasmático pode variar, e a avaliação da sobrecarga de volume geralmente é verificada pela presença de edema periférico e peso corporal. Por outro lado, também queríamos demonstrar que o ecocardiograma, mesmo nessas circunstâncias, tinha um alto poder de discriminar quem realmente tinha diagnóstico confirmado de hipertensão pulmonar, apesar das divergências nas medidas diretas.

Quanto ao intervalo entre o ecocardiograma inicial e o cateterismo cardíaco direito no presente estudo – realizado nas condições da prática clínica habitual – vale ressaltar que discordâncias entre essas medidas também foram relatadas por Fisher et al. em seu estudo realizado em condições nas quais os procedimentos foram realizados com intervalo de apenas uma hora.^1^Por outro lado, Farber et al. relataram resultados semelhantes ao analisarem dados do grande registro norte-americano de hipertensão pulmonar – REVEAL, onde o intervalo entre os exames foi ainda maior, com no máximo 12 meses. Neste estudo, usando o CCD como referência, o ECO foi considerado impreciso em 57,4% das estimativas da PSAP (>10 mmHg acima ou abaixo do CCD) e em 36,5% das estimativas médias da PAD (>5 mmHg acima ou abaixo do CCD). Vale ressaltar que a correlação entre o RHC inicial e os parâmetros ECO iniciais foi semelhante quer os exames tenham sido realizados no mesmo dia, no mesmo mês, ou 1 a 3 meses, 3 a 6 meses ou 6 a 12 meses.^2^

Também concordamos sobre o efeito da anemia e da deficiência de ferro na hipertensão pulmonar. Ressalta-se que o nível médio de hemoglobina da série apresentada foi de 13,3 g/dL ± DP=0,9 (dados não apresentados no artigo), sugerindo que no presente estudo esse efeito provavelmente não foi significativo.

Concluindo, gostaríamos de enfatizar novamente que, apesar das divergências observadas em termos absolutos das medidas de pressão atrial direita e pressão sistólica da artéria pulmonar, a indicação das medidas hemodinâmicas e o diagnóstico final de hipertensão pulmonar não foram afetados, uma vez que outros critérios relevantes para o manejo diagnóstico foram adotados, alertando-nos para o fato de que a estimativa ecocardiográfica absoluta da pressão sistólica da artéria pulmonar muitas vezes não é suficiente para reduzir a probabilidade de diagnóstico dessa condição. O volume e o nível de hemoglobina do paciente devem ser adequadamente avaliados no contexto do diagnóstico de hipertensão pulmonar.

## References

[1] Fisher MR, Forfia PR, Chamera E, Housten-Harris T, Champion HC, Girgis RE (2009). Accuracy of Doppler Echocardiography in the Hemodynamic Assessment of Pulmonary Hypertension. Am J Respir Crit Care Med.

[2] Farber HW, Foreman AJ, Miller DP, McGoon MD (2011). REVEAL Registry: Correlation of Right Heart Catheterization and Echocardiography in Patients with Pulmonary Arterial Hypertension. Congest Heart Fail.

# Reply

**Authors:** Camila Farnese Rezende, Eliane Viana Mancuzo, Maria do Carmo Pereira Nunes, Ricardo Amorim Corrêa

**Dear Editor,**

We would like to thank you for the very pertinent and enlightening comments from the reader about the variables that have the potential to explain disagreements between echocardiographic (ECHO) estimates of right atrial (RAP) and pulmonary artery systolic pressures (PASP) and those obtained by direct catheterization of the right heart chambers and pulmonary artery (RHC).

We agree with the comments. The status of intravascular volume can vary over a three-month interval, especially in outpatients. It is well known that this assessment by ECHO is complex, depending on the parameters of the inferior vena cava and even invasive measurement of central venous pressure, which is not routinely done in clinical practice. Although this is a limitation of the study, in clinical practice, plasma volume can vary, and the assessment of volume overload is usually verified by the presence of peripheral edema and body weight. On the other hand, we also wanted to demonstrate that the echocardiogram, even subject to these circumstances, had a high power to discriminate who actually had a confirmed diagnosis of pulmonary hypertension, despite the disagreements in the direct measurements.

With regard to the interval between the initial echocardiogram and the right heart catheterization in the present study – performed under the conditions of usual clinical practice – it is worth noticing that disagreements between these measurements were also reported by Fisher et al. in their study carried out under conditions in which the procedures were done with an interval of just one hour.^1^On the other hand, Farber et al. reported similar results when they analyzed data from the large North American pulmonary hypertension registry – REVEAL, where the interval between tests was even longer, with a maximum of 12 months. In this study, using RHC as the reference, ECHO was considered inaccurate in 57.4% of PASP estimates (>10 mm Hg higher or lower than RHC) and in 36.5% of mean RAP estimates (>5 mm Hg higher or lower than RHC). It is noteworthy that the correlation between initial RHC and initial ECHO parameters was similar whether the tests were performed on the same day, within the same month, or 1 to 3 months, 3 to 6 months, or 6 to 12 months.^2^

We also agree on the effect of anemia and iron deficiency on pulmonary hypertension. It should be noted that the mean hemoglobin level of the series presented was 13.3 g/dL ± SD=0.9 (data not presented in the article), suggesting that in the present study, this effect was probably not significant.

In conclusion, we would like to re-emphasize that, despite the disagreements observed in absolute terms of the measurements of right atrial pressure and pulmonary artery systolic pressure, the indication for hemodynamic measurements and the final diagnosis of pulmonary hypertension were not affected once other criteria relevant to diagnostic management were adopted, alerting us to the fact that the absolute echocardiographic estimate of pulmonary artery systolic pressure is often not enough to reduce the probability of diagnosing this condition. The patient's volume and hemoglobin level must be properly assessed in the context of the diagnosis of pulmonary hypertension.
